# Supplementary material for: Learning curve and surgical outcome of robotic assisted colorectal surgery with ERAS program
Source: Sci Rep. 2022 Nov 29;12:20566. doi: 10.1038/s41598-022-24665-w (PMC9709162; doi:10.1038/s41598-022-24665-w)
Supplement: Supplementary file 2 — Supplementary Information 2. [file 41598_2022_24665_MOESM2_ESM.zip › RAL Raw data-Ñ[▒K/119/HS_ñw╝╨░O▒Kñσ.pdf]

正式摘要(電子簽章：000000227600923)		
W83 / W83 ☐ 英文標題(注意:無英文姓名-請補病人基本檔!)	☐【讀取】憑證	

臺中榮民總醫院
出院病歷摘要

轉入號： 轉出號：

[redacted]

**(8)**流水編號	
**(9)**入院日期	110/10/13 (2021)
**(10)**轉科日期	
**(11)**出院日期	110/10/19 (2021) 住院天數計 6 日

診斷

- 入院 :1.Rectal cancer s/p CCRT, regression
  2.pulmonary nodule, LLL, s/p wedge resection on 2021.9.8, benign
  3.hypertension
- 出院 :1.Rectal cancer s/p CCRT, regression s/p robotic APR on 2021/10/14
  2.pulmonary nodule, LLL, s/p wedge resection on 2021.9.8, benign
  3.hypertension

主訴

- constipation, bloody stool since last year

病史

- This 63 years old female patient has past history of hypertension. She complained perianal pain might due to herpes zoster during last September to November. After recovery, pain during defecation didn't relieve. Constipation and intermiitent bloody stool was also found. She came to 門諾 H. for help on March 2021. Colonoscopy was done and rectal tumor at dentale line was found that pathology report showed adenocarcinoma. She started CCRT. Follow-up PET on Augest showed a nodule at left lower lobe of lung. Operation with wedge resection was done on 2021.9.8 that no malignancy was found in pathology. This time, she was admitted for surgical intervention.

體檢發現

-
  - General appearance :
    - easy looking , conscious clear , without cardiopulmonary distress
    - Vital signs自動帶入date 體溫(BT) 脈搏(P) 呼吸(R) 血壓(BP) 身高(BH) 體重(BW) BMI
      20211013 35.9℃ 77次/分 18次/分 168/79mmHg 152cm 48kg 20.8
  - HEENT :
    - Eyes: no anemic conjunctiva, no icteric sclera, normal EOM
    - ENT: no otorrhea, no rhinorrhea, no erythematous mucosa, no injected tonsil, no deformity
  - Neck: supple, freely movable, trachea midline,no deformity, no engorged jugular vein , no palpable thyroid or lymph node enlargement
  - Chest:
    - Inspection: Grossly normal, symmetric and fully expansion, no accessory muscle used, no operation scar, no spider angiomata, no gynecomastia
    - Palpation: no palpable mass or nodule, no subcutaneous emphysema, no tenderness
    - Ausculation: clear breathing sound
  - Heart:
    - Percussion: normal heart size
    - Ausculation: regular heart beat, normal S1, S2, no gallop, no murmur
  - Abdomen:
    - Inspection: in
      - flat contour, symetrically, no scar, no stria, no dilated veins, no rashes
    - Ausculation: normoactive bowel sound, no abdominal bruits
    - palpation: soft, no muscle guarding or rigidity, no local tenderness, no rebounding pain, no palpable mass, no palpable pulsation

-
  - Percussion: no shifting dulllness, no hepatomegaly, no splenomagaly
-

Peripheral pulsation :	CA	BA	RA	FA	PA	PTA	DPA
Right side :	++	++	++	++	++	++	++/+++
Left side :	++	++	++	++	++	++	++/+++

- Genito-anus: no tenderness or palpable mass lesion or rectal shelf, normal external genitalia, normal anal tone, brown stool

CRS CONDITION

- CEA: 1.98 ng/ml
-
  - Lung

手術日期及方法

- 手術日期及方法20211014 Robot Assisted Low Anterior Resection

住院治療經過

- After admission, operation with robotic APR was done on 10/14. We let her try water and diet under ERAS protocol. However, less urine output and vomited twice was found on POD2 and KUB showed dilated colon. Thus, IVF was prescribed. Much urine output was noted on POD3. Drainage tube was removed on POD4. Under the stable condition, she was discharged on 10/19.

合併症

- nil

一般檢查

- 累積報告：

DATE	TIME	ALB	TP	BIL.T	BIL.D	ALKP	AST	ALT	LDH	AMY	LIPASE	NH3	Ferritin	UA
1101013	1746		6.9											5.9
1101014	1951			0.60	0.14		27	22						
1101017	0900	3.5		0.7	0.2			12						

DATE	TIME	WBC	RBC	HGB	HCT	MCV	PLT	BLST	NEUT	BAND	SEG	LYM	MON	EOS	BAS
1101014	1950	7100		9.2	28.6		134				91.0	7.0	2.0	0.0	0.0
1101017	0850	4920	3.79	9.0	28.3	74.7	129		69.5			25.0	4.1	1.2	0.2

DATE	TIME	NA	K	CL	BUN	CREAT	eGFR	CA	Mg	P	CRP	ESR	PCT	D-Dimer	LACTATE
1101013	1746										0.024				
1101014	1951	142	3.6		8	0.66	96.14	8.8							19.7
1101017	0804														6.8
1101017	0900	143	3.4		5	0.66	96.14	8.0							

DATE	TIME	GLUCOSE	HbA1C	CHOL	TG	HDL	LDL	KETONE	CK	CKMB	TRO-T	PT-P	APTT-P	NT-pro BNP
1101013	1746			242	141	87	139							

DATE	TIME	PH	BEB	SCVO2	PO2	HCO3	HGB	PCO2	TCO2
1101014	1952	7.391	1.7	98.9	122.1	27.0		45.6	28.4

特殊檢查

病理報告

其它

出院時情況

- 3.改門診治療

出院指示

藥名	劑量	劑量單位	用法	頻次	數量	數量單位	現況	天數	單位
Acetal tab 500mg	1	TAB	PO	Q6HPRN	56	TAB	TH	14	天
Esomeprazole tab	1	TAB	PO	QDAC	14	TAB	TH	14	天
Flurbiprofen 100mg	1	TAB	PO	BIDPRN	28	TAB	TH	14	天
Mag. Oxide tab	2	TAB	PO	TID	84	TAB	TH	14	天
MOSAD tab 5mg	1	TAB	PO	TID	42	TAB	TH	14	天

出院計劃

主治醫師 2062F 蔣鋒帆 / 住院醫師 2331F 陳凱祥

正式摘要(電子簽章：000000227600923)

		W83 / W83				

doctorRank:B
